# Lessons from public health policies contributing to Brazil's lowest maternal mortality ratio in 2022: a short communication

**DOI:** 10.1590/1806-9282.20251688

**Published:** 2025-12-15

**Authors:** Raphael Câmara Medeiros Parente, Marcelo Antônio Cartaxo Queiroga Lopes, Lana de Lourdes Aguiar Lima, Patrícia Marçal

**Affiliations:** 1 Ministry of Health, Primary Health Care – Rio de Janeiro (RJ), Brazil.; 2 Ministry of Health – João Pessoa (PB), Brazil.; 3 Ministry of Health, Department of Maternal and Child Health – João Pessoa (PB), Brazil.

## INTRODUCTION

The maternal mortality ratio (MMR), defined as maternal deaths per 100,000 live births, is a key indicator of health system quality and equity^1^. Globally, 99% of maternal deaths occur in developing countries, with a lifetime risk 120 times higher in low-income nations compared to high-income ones^1^. In Brazil, regional disparities, particularly in the North and Northeast, have long challenged maternal health efforts^2,3^. The COVID-19 pandemic intensified these issues, with MMR rising to 117.4/100,000 in 2021^4^. Between 2019 and 2022, the Ministry of Health (MoH) implemented policies such as the Maternal and Child Health Care Network (RAMI), launched via Ordinance GM/MS No. 715 in 2022^5^, and Cuida Mais Brasil, which aimed to integrate gynecologists into primary care^6^. These initiatives were supported by substantial government investments, reaching approximately 12 billion BRL, aimed at strengthening maternal and child health services^7^. Prioritization of pregnant women in the National Immunization Plan resulted in the vaccination of over 2.3 million women by 2022^8^. This study investigates the hypothesis that these policies, together with broader investments in maternal and child health, contributed to the reduction of the MMR to 57.7/100,000 in 2022. The objective is to analyze the relationship between the MoH policies implemented between 2020 and 2022 and the lowest MMR ever recorded in Brazil in 2022, considering the context of the COVID-19 pandemic.

## METHODS

This descriptive study used secondary data from the Mortality Information System (SIM) and Live Birth Information System (SINASC) via DATASUS (January–May 2023), covering 2002–2022, and reviewed 2020–2022 public health policies (e.g., RAMI, Cuida Mais Brasil). The MMR was calculated as maternal deaths (SIM, ICD-10 O00–O99, excluding incidental causes) per 100,000 live births (SINASC), with corrections since 2009 by rechecking deaths of women aged 10–49 for missed pregnancy-related cases. Prenatal care coverage was the percentage of pregnant women with one prenatal consultation (SINASC). MMR was analyzed across Brazil's five macro-regions and selected state capitals (e.g., Curitiba, Belo Horizonte, Florianópolis, Boa Vista, Macapá, Porto Velho) for reliable data and MMR extremes in Table 1. Investments were derived from 2020–2022 ministerial ordinances (Nos. 2.222/2020, 3.186/2020, 731/2021, 894/2021, 715/2022, 2.228/2022, 937/2022) via the Virtual Health Library, verified by Portal da Transparência and Plataforma Mais Brasil. No Ethics Committee approval was needed. Descriptive statistics summarized MMR, deaths, births, and variations. MMR trends used Joinpoint-inspired segmented linear regression (2002–2019, 2020–2022), with log-transformed MMR values. APC was calculated as APC=(e^β-1)×100, where β is the regression slope (p<0.05).

**Table 1:** Maternal mortality ratio in selected capitals, 2022.

Capital	Maternal deaths	Live births	MMR (per 100,000 live births)*
Curitiba (PR)	5	30,675	16.3
Belo Horizonte (MG)	12	48,387	24.8
Florianópolis (SC)	7	24,055	29.1
Boa Vista (RR)	15	9,047	165.8
Macapá (AP)	13	8,747	148.6
Porto Velho (RO)	11	9,061	121.4

* Continuous variable, calculated as (maternal deaths/live births)×100,000.

MMR: maternal mortality ratio.

## RESULTS

In 2022, the MMR in Brazil reached 57.7 per 100,000 live births (1,368 maternal deaths among 2,561,922 live births). This represents a 51% reduction compared to the MMR of 117.4 per 100,000 live births in 2021.

**Figure 1:**
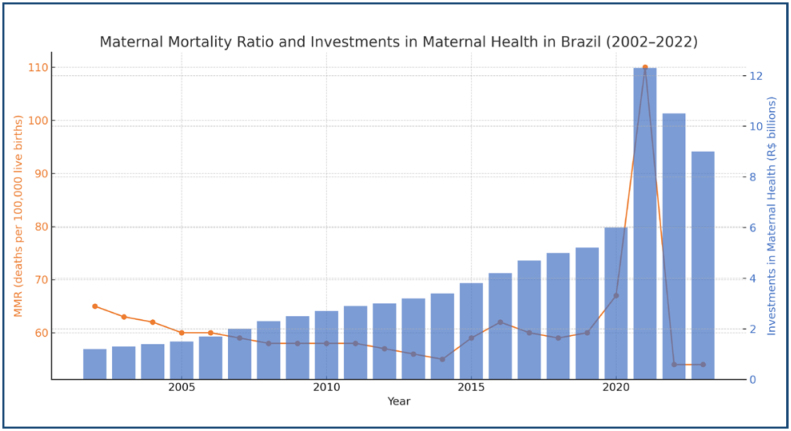
Trends in maternal mortality ratio and annual investments in maternal health (2002–2022).

Joinpoint-inspired segmented linear regression analysis revealed distinct trends in MMR over the study period. Prior to the COVID-19 pandemic (2002–2019), the MMR exhibited a declining trend, with an APC of -1.64 (p<0.001). Following the onset of the pandemic in 2020, the analysis indicated a post-2020 "recovery" period (2020–2022), with an APC of -12.11 (p=0.766) (Figure 1).

Figure 1 displays the trends in MMR and annual investments in maternal health from 2002 to 2022, providing a visual representation of the temporal relationship between these two variables. The graph shows MMR on the primary y-axis and investment (in BRL billions) on the secondary y-axis, allowing for a direct comparison of their trajectories over time. The figure illustrates a declining MMR trend from 2002 to 2019, followed by increases in 2020 and 2021, and a subsequent decrease in 2022.

Table 2 presents the MMR by region in Brazil for 2022, allowing for a clear comparison of regional disparities in maternal mortality. Regions were defined according to the standard geographic divisions of Brazil (North, Northeast, South, Southeast, and Center-West). The data reveals significant differences in MMR across regions, with the North exhibiting the highest MMR (82.0 per 100,000 live births) and the South exhibiting the lowest (40.0 per 100,000 live births).

**Table 2:** Maternal mortality ratio by region in Brazil, 2022.

Region	Maternal deaths	Live births	MMR (per 100,000 live births)*
South	140	350,000	40.0
Southeast	540	1,073,758	50.3
Center-West	110	192,982	57.1
Northeast	470	698,368	67.3
North	180	219,512	82.0

* Continuous variable, calculated as (maternal deaths/live births)×100,000.

MMR: maternal mortality ratio.

Table 1 shows the MMR in selected capital cities, chosen to highlight the range of MMR values across different urban centers. The selection of capital cities aimed to include both those with the lowest and highest MMRs in the country. The table demonstrates substantial variation in MMR across these cities, ranging from a low of 16.3 per 100,000 live births in Curitiba to a high of 165.8 per 100,000 live births in Boa Vista.

## DISCUSSION

The 2022 MMR of 57.7/100,000, Brazil's lowest since 2002, reflects a 51% drop from 117.4/100,000 in 2021. The prepandemic decline (APC=-1.64, p<0.001) aligns with regional trends, while the post-2020 recovery (APC=-12.11, p=0.766) suggests a potential positive shift linked to Ministry initiatives. The appointment of qualified professionals, such as obstetrician-gynecologists, to key roles like the Secretary of Primary Health Care (SAPS) and Women's Health Coordination ensured technical expertise. Regular meetings between the Minister of Health and the Secretary of Primary Health Care, together with the technical team, enhanced accountability and resource allocation.

Extraordinary credits and ordinances (e.g., Nos. 2,222/2020, 731/2021, 894/2021, 3,186/2020) directed billions to maternity hospitals and prenatal care, with investments peaking at ∼12 billion BRL. These high investments in maternal and child health likely contributed to the reduction in MMR, particularly in regions with previously limited resources. The Maternal and Child Health Care Network (RAMI), launched in 2022 via Ordinance GM/MS No. 715, represented a positive evolution of maternal care frameworks, increasing funding for low-risk maternity hospitals and high-risk pregnancy clinics, and improving access to quality care^5^. Cuida Mais Brasil, with a 2022 budget of R$170 million, integrated gynecologists into primary care, addressing inadequate prenatal care, a key determinant of maternal mortality^6^. Cuida Mais Brasil represented a novel approach to maternal care by integrating gynecologists into primary care teams, addressing a long-standing gap in access to specialized services for many women. Over 2.3 million pregnant and postpartum women were vaccinated against COVID-19 by 2022, reducing severe outcomes^8^. Prenatal care coverage reached 78.9%, driven by Previne Brasil indicators. This expansion of prenatal care services, promoted and supported by the MoH during the 2019–2022 period, likely contributed to improved maternal health outcomes.

Partnerships with scientific societies have led to the development of evidence-based guidelines, such as the Manual of Recommendations for Care of Pregnant and Postpartum Women^9^ and the High-Risk Pregnancy Manual^10^, which have been disseminated across the SUS network. These efforts coincided with a historic low in adolescent pregnancy rates, linked to comprehensive sexual education and non-abortive family planning^11^.

While the segmented regression analysis did not demonstrate a statistically significant change in the trend of MMR after 2020 (p=0.766), it is important to note that the absolute reduction in MMR in 2022 is substantial and clinically meaningful. The lack of statistical significance may be due to the relatively short time period (2020–2022) included in the postintervention analysis, which limits the power to detect changes in the trend. Although an association between the policies and MMR decline was observed, this does not establish causation. Other unmeasured factors may have contributed to the observed decline. Social and behavioral changes driven by the COVID-19 pandemic could have contributed to this decline^12,13^. For example, some individuals may have chosen not to conceive or delay pregnancies out of fears or uncertainties related to the virus. Additionally, women of higher reproductive age might have opted against further pregnancies due to pandemic-related risks^14^.

Limitations include SIM underreporting, preliminary 2022 data, and the potential for confounding variables, which limit our ability to attribute the MMR decline only to the implemented policies definitively. The post-2020 variability (p=0.766) may reflect policy lag effects. RAMI's 2023 revocation poses a risk. Further research, employing more rigorous causal inference methods, is needed to disentangle the specific contributions of these policies from other concurrent factors.

## CONCLUSION

The decline in Brazil's MMR in 2022 coincided with the implementation of MoH policies focused on strengthening maternal and child health care. These included the RAMI and Cuida Mais Brasil programs and targeted investments in prenatal and hospital care. Although causality cannot be confirmed, the temporal association suggests a potential contribution of these initiatives to improved maternal outcomes. Continued monitoring and sustained investments are essential to consolidate these gains and reduce regional disparities.

## Data Availability

The datasets generated and/or analyzed during the current study are available from the corresponding author upon reasonable request.
